# Cytokines TNF-*α*, IL-6, IL-17F, and IL-4 Differentially Affect Osteogenic Differentiation of Human Adipose Stem Cells

**DOI:** 10.1155/2016/1318256

**Published:** 2016-09-07

**Authors:** Angela P. Bastidas-Coral, Astrid D. Bakker, Behrouz Zandieh-Doulabi, Cornelis J. Kleverlaan, Nathalie Bravenboer, Tim Forouzanfar, Jenneke Klein-Nulend

**Affiliations:** ^1^Department of Oral Cell Biology, Academic Centre for Dentistry Amsterdam (ACTA), University of Amsterdam and Vrije Universiteit Amsterdam, MOVE Research Institute Amsterdam, Amsterdam, Netherlands; ^2^Department of Dental Materials Science, Academic Centre for Dentistry Amsterdam (ACTA), University of Amsterdam and Vrije Universiteit Amsterdam, MOVE Research Institute Amsterdam, Amsterdam, Netherlands; ^3^Department of Clinical Chemistry, VU University Medical Center, MOVE Research Institute Amsterdam, Amsterdam, Netherlands; ^4^Department of Oral and Maxillofacial Surgery, VU University Medical Center, MOVE Research Institute Amsterdam, Amsterdam, Netherlands

## Abstract

During the initial stages of bone repair, proinflammatory cytokines are released within the injury site, quickly followed by a shift to anti-inflammatory cytokines. The effect of pro- and anti-inflammatory cytokines on osteogenic differentiation of mesenchymal stem cells is controversial. Here, we investigated the effect of the proinflammatory cytokines TNF-*α*, IL-6, IL-8, and IL-17F and the anti-inflammatory cytokine IL-4 on proliferation and osteogenic differentiation of human adipose stem cells (hASCs). hASCs were treated with TNF-*α*, IL-6, IL-8, IL-17F, or IL-4 (10 ng/mL) for 72 h mimicking bone repair. TNF-*α* reduced collagen type I gene expression but increased hASC proliferation and ALP activity. IL-6 also strongly enhanced ALP activity (18-fold), as well as bone nodule formation by hASCs. IL-8 did not affect proliferation or osteogenic gene expression but reduced bone nodule formation. IL-17F decreased hASC proliferation but enhanced ALP activity. IL-4 enhanced osteocalcin gene expression and ALP activity but reduced RUNX2 gene expression and bone nodule formation. In conclusion, all cytokines studied have both enhancing and reducing effects on osteogenic differentiation of hASCs, even when applied for 72 h only. Some cytokines, specifically IL-6, may be suitable to induce osteogenic differentiation of mesenchymal stem cells as a strategy for enhancing bone repair.

## 1. Introduction

The treatment of critical-size cranial defects is still a significant challenge. These defects can result from craniectomy due to trauma and tumors. Despite complications related to the harvesting procedure, such as haemorrhage, nerve and vascular lesions, and prolonged or chronic postoperative pain, bone grafts are still considered the gold standard in the reconstruction of craniomaxillofacial skeletal defects [1, 2]. Yet, bone tissue engineering techniques, including the use of mesenchymal stem cells (MSCs), scaffolds, and inductive factors such as cytokines, can also be used as a strategy to repair critical-size cranial defects [3].

The physiological process of bone repair implicates the formation of a haematoma followed by an inflammatory response, which has been demonstrated to play a crucial role in early fracture repair [4, 5]. During the inflammatory phase, different cytokines are released at the injury site to aid the recruitment of mesenchymal progenitor cells, followed by replacement of the haematoma with granulation tissue [6]. Known proinflammatory cytokines, such as tumor necrosis factor-*α* (TNF-*α*), interleukin-6 (IL-6), and interleukin-8 (IL-8), can be detected in an early stage of fracture healing. High levels of TNF-*α* have been detected within the first 24 hours after bone injury in a mouse tibia fracture model [7]. In patients with hip fractures, expression of IL-6 and IL-8 has been shown to be elevated within the first 24 to 72 hours after bone injury [8, 9]. TNF-*α* and IL-6 are secreted by macrophages and T-cells, and IL-6 is also secreted by osteoblasts [10]. TNF-*α* is known to promote the recruitment of MSCs and osteoblasts [7]. IL-6 has been shown to stimulate osteoblast differentiation [10]. In addition, IL-6 positively influences the mitogen-activated protein kinase signaling cascade, which is essential for bone formation in human bone marrow MSCs (BMSCs) [11]. The hypoxia-regulated cytokine IL-8 is upregulated during haematoma formation [9]. Interleukin-17F (IL-17F), a cytokine secreted by T-helper cell 17 (Th17) subset, has been shown to be expressed during the early phase of fracture healing, that is, 72 hours after fracture in mice, using immunohistochemistry [12]. Moreover, IL-17F stimulates osteoblast maturation* in vitro* [12, 13]. Recently, IL-17F has been shown to strongly induce osteogenic differentiation of MSCs [14].

Following the initial inflammatory response, a shift from proinflammatory to anti-inflammatory cytokines occurs, which is crucial for the repair process [15]. The T-helper 2 (Th2) cytokines IL-4, IL-10, and IL-13 belong to the family of anti-inflammatory cytokines that play an important role in inflammatory and immune responses [16, 17]. In particular, IL-4 inhibits bone resorption [18, 19], and its depletion can lead to a reduction in cortical bone mass in adult male mice [20]. In addition, IL-4 enhances osteogenesis by cocultures of proinflammatory M1 macrophages with preosteoblastic MC3T3 cells by modulating the M1 macrophage phenotype towards M2 [21].

Cytokines may affect the osteogenic differentiation of MSCs, besides their role in the immune response initiated upon injury. Both positive and negative effects of cytokines on osteogenic differentiation of MSCs have been reported, which might be related to the kinetics of their application [22, 23]. Whether IL-4, TNF-*α*, IL-6, IL-8, and/or IL-17F application for a duration of 72 hours, mimicking the* in vivo* situation, affects proliferation and osteogenic differentiation of MSCs is still unclear. A better understanding of the inflammatory phase during bone repair is crucial to exploit the regenerative potential of MSCs. Therefore, the aim of this study was to investigate whether a short exposure to proinflammatory and anti-inflammatory cytokines, known to be released during bone fracture, modulates proliferation and/or osteogenic differentiation of MSCs. We stimulated human adipose stem cells (hASCs) with the proinflammatory cytokines TNF-*α*, IL-6, IL-8, and IL-17F and the anti-inflammatory cytokine IL-4 for 72 hours. Proliferation was assessed by KI67 gene expression and DNA quantification. Osteogenic differentiation of hASCs was studied by analysis of gene expression of RUNX2, collagen type 1 (COL1), and osteocalcin (OC), as well as alkaline phosphatase (ALP) activity and bone nodule formation.

## 2. Materials and Methods

### 2.1. Adipose Tissue Donors

Subcutaneous adipose tissue samples were harvested from abdominal wall resections of five healthy female donors (age range: 33–54 years, mean: 47 years), who underwent elective plastic surgery at the Tergooi Hospital Hilversum and a clinic in Bilthoven, The Netherlands. The Ethical Review Board of the VU Medical Center, Amsterdam, The Netherlands, approved the protocol (number 2016/105) and informed consent was obtained from all patients.

### 2.2. Isolation and Culture of hASCs

Isolation, characterization, and osteogenic differentiation capacity of hASCs have been reported previously by our group [24]. For the isolation of hASCs, adipose tissue was cut into small pieces and enzymatically digested with 0.1% collagenase A (Roche Diagnostics GmbH, Mannheim, Germany) in phosphate-buffered saline (PBS) containing 1% bovine serum albumin (Roche Diagnostics GmbH) under continuous shaking conditions for 45 min at 37°C. Next a Ficoll® density-centrifugation step (Lymphoprep™; 1,000 g, 20 min, *ρ* = 1.077 g/mL Ficoll, osmolarity 280 ± 15 mOsm; Axis-Shield, Oslo, Norway) was performed, and the cell-containing interface was harvested and resuspended in Dulbecco's modified Eagle's medium (Life Technologies™ Europe BV, Bleiswijk, The Netherlands). hASCs were counted and stored in liquid nitrogen. Cryopreserved hASCs from the different donors were pooled and cultured in *α*-Minimum Essential Medium (*α*-MEM; Gibco, Life Technologies, Waltham, MA, USA) with 1% penicillin, streptomycin, and fungizone (PSF; Sigma, St. Louis, MO, USA), 10 IU/mL heparin (LEO Pharma A/S, Ballerup, Denmark), and 2% human platelet lysate, at 37°C in 5% CO_2_ in air. The medium was refreshed every 3 days. When near confluent (90%), hASCs were harvested by adding 0.25% trypsin (Gibco, Invitrogen, Waltham, MA, USA) and 0.1% ethylenediaminetetraacetic acid (Merck, Darmstadt, Germany) in PBS at 37°C. ASCs were stored in liquid nitrogen until further use. For experiments, hASCs were thawed and seeded at 0.5 × 10^6^ cells in T-175 cm^2^ culture flasks (Greiner Bio-One, Kremsmünster, Austria) in *α*-MEM containing 1% PSF, 10 IU/mL heparin, and 2% human platelet lysate, at 37°C in 5% CO_2_ in air. In all experiments, hASCs at passage 2 (P2) were used. Medium was changed every 3 days.

### 2.3. Platelet Lysate

Pooled platelet products from five donors were obtained from the Bloodbank Sanquin (Sanquin, Amsterdam, The Netherlands) and contained approximately 1 × 10^9^ platelets per mL [25]. Platelet lysate was obtained by lysing the platelets through temperature shock at −80°C. For usage, platelet lysate was thawed and centrifuged at 600 g for 10 min to eliminate remaining platelet fragments. The supernatant was added at 2% (v/v) to the medium or stored at 4°C until usage within 1 week.

### 2.4. Stimulation of hASCs with Proinflammatory and Anti-Inflammatory Cytokines

hASCs (1 × 10^4^ cells/cm^2^) were seeded in 24-well plates and cultured in *α*-MEM containing 1% PSF, 10 IU/mL heparin, and 2% human platelet lysate, at 37°C in 5% CO_2_ in air. hASCs were allowed to attach for 24 h before stimulation with cytokines. After cytokine stimulation, the medium was replaced with osteogenic medium (OM), consisting of *α*-MEM containing 1% PSF, 10 IU/mL heparin, 2% human platelet lysate, 50 *μ*M ascorbic acid-2-phosphate (vitamin C; Sigma, St. Louis, MO, USA), 5 mM *β*-glycerophosphate (*β*GP; Sigma), and 10 nM 1,25-(OH)_2_ vitamin D_3_ (Sigma). Recombinant human TNF-*α* (R&D Systems, Minneapolis, MN, USA), recombinant human IL-4 (R&D Systems), recombinant human IL-6 (R&D Systems), recombinant human IL-6R*α* (R&D Systems), recombinant human IL-8 (R&D Systems), and recombinant human IL-17F (R&D Systems) were added to the OM at 10 ng/mL and incubated for 72 h at 37°C in 5% CO_2_ in air. Then, the medium was changed to OM without cytokines and was replaced every 3 days. hASCs were harvested at 6 and 48 h (early time points) and at 4, 7, and 14 days (late time points) to assess proliferation and osteogenic differentiation of hASCs.

### 2.5. Cell Proliferation

hASCs cultured for 48 h, 4 days, and 7 days with proinflammatory and anti-inflammatory cytokines were washed with PBS, and CyQuant lysis buffer was added. DNA content, as a measure for cell number, was determined using the CyQuant Cell Proliferation Assay Kit (Molecular Probes, Leiden, The Netherlands). Absorption was read at 485 nm excitation and 528 nm emission in a microplate reader (Synergy HT® spectrophotometer; BioTek Instruments Inc., Highland Park, Winooski, VT, USA).

### 2.6. RNA Isolation and Real-Time RT-PCR

Total RNA was isolated from hASCs using TRIzol® reagent (Invitrogen, Carlsbad, CA, USA), according to the manufacturer's instructions. Total RNA concentration and quality were determined using a Synergy HT spectrophotometer. RNA was reverse-transcribed to cDNA using a RevertAid™ First Strand cDNA Synthesis Kit (Fermentas, St. Leon-Rot, Germany) according to the manufacturer's instructions. Real-time PCR was performed using SYBR® Green I Mastermix (Roche Diagnostics, Mannheim, Germany) in a LightCycler® 480 (Roche Diagnostics, Basel, Switzerland). Every PCR reaction was prepared with 3 *μ*L PCR-H2O, 0.5 *μ*L forward primer (1 *μ*M), 0.5 *μ*L reverse primer (1 *μ*M), 5 *μ*L LightCycler 480 SYBR Green I Mastermix (Roche Diagnostics, Mannheim, Germany), and 1 *μ*L cDNA in a final volume of 10 *μ*L. Based on BestKeeper [26], the values obtained were normalized to YWHAZ and UBC housekeeping genes. Real-time PCR was used to assess expression of the following genes: KI67, RUNX2, COL1, and osteocalcin. All primers used were from Life Technologies. The primer sequences are listed in Table 1. mRNA preparations from human bone were used as a reference and internal control in each assay.

### 2.7. Alkaline Phosphatase Activity

hASCs cultured for 48 h, 4 days, and 7 days with proinflammatory and anti-inflammatory cytokines were lysed with CyQuant lysis buffer. ALP activity was measured in the cell lysate using 4-nitrophenyl phosphate disodium salt (Merck, Darmstadt, Germany) at pH 10.3 as a substrate for ALP, according to the method described by Lowry [27]. The absorbance was read at 405 nm with a Synergy HT spectrophotometer. ALP activity was expressed as *μ*M per ng DNA.

### 2.8. Mineralization

Matrix mineralization was analyzed by alizarin red staining after incubation of hASCs with proinflammatory and anti-inflammatory cytokines at day 14 by using 1% Alizarin Red S (pH 4.1; Sigma-Aldrich, St. Louis, MO, USA) in water as described earlier [28]. Briefly, hASCs were fixed with 10% formaldehyde for 15 min and rinsed with deionized water before adding 350 mL of 1% Alizarin Red S solution per well. After incubation for 15 min at room temperature, the cells were washed with deionized water. Cells differentiating into osteoblasts show mineralized matrix deposition, producing bright red nodules.

### 2.9. Statistical Analysis

Values are provided as mean ± SD. Differences between two groups were tested for statistical significance using paired* t*-test. Analysis of variance (ANOVA) was used to compare data between three or more groups, with application of Dunnett's multiple comparison test to compare with untreated controls. A *p* value < 0.05 was considered significant. Statistical analysis was performed using GraphPad Prism 5.4 (GraphPad Software, San Diego, CA, USA).

## 3. Results

### 3.1. TNF-*α*, but Not IL-6, IL-8, IL-17F, or IL-4, Stimulates hASCs DNA Content

**Figure 1:**
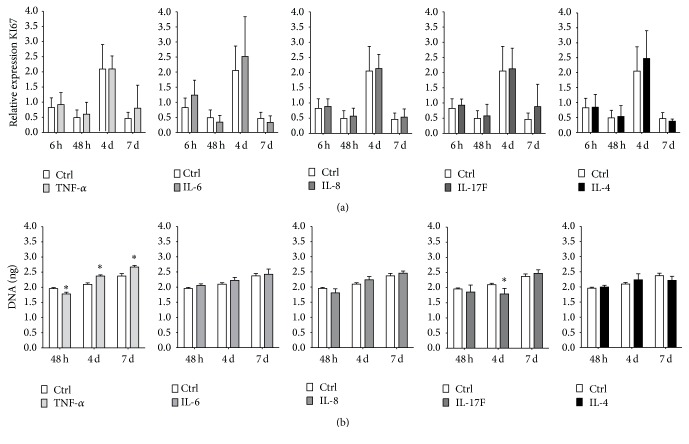
Comparative analysis of the effect of pro- and anti-inflammatory cytokines on hASC proliferation. ASCs were stimulated for 72 h with proinflammatory cytokines TNF-*α*, IL-6, IL-8, and IL-17F and the anti-inflammatory cytokine IL-4 (10 ng/mL). (a) Gene expression of proliferation marker KI67 at 6 h, 48 h, and days 4 and 7. No significant effects of cytokines on KI67 expression were found, *n* = 7. (b) DNA content at 48 h, day 4, and day 7. TNF-*α* decreased DNA content at 48 h but increased DNA content at days 4 and 7. IL-17F decreased DNA content at day 4. *n* = 3, results are mean ± SD. ^*∗*^Significant effect of cytokine treatment, *p* < 0.05.

DNA content and gene expression of the proliferation marker KI67 were analyzed to assess whether proinflammatory and anti-inflammatory cytokines affect hASCs proliferation. All cytokines did not affect KI67 gene expression compared with untreated cultures at 48 h, day 4, or day 7 (Figure 1(a)). TNF-*α* significantly decreased DNA content by 1.1-fold at 48 h, but it increased DNA content by 1.1-fold at days 4 and 7 compared to untreated controls (Figure 1(b)). IL-17F decreased DNA content by 1.2-fold at day 4 (Figure 1(b)). All other cytokines did not affect DNA content compared with untreated cultures at 48 h, day 4, or day 7 (Figure 1(b)).

### 3.2. Cytokines Exerted Various Effects on Gene Expression of Osteogenic Markers in hASCs

**Figure 2:**
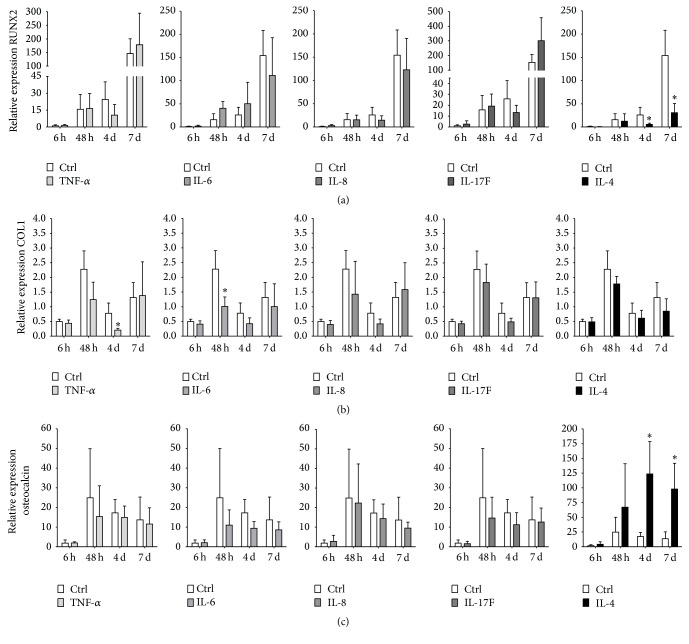
Comparative analysis of the effect of pro- and anti-inflammatory cytokines on osteogenic differentiation of hASCs. ASCs were stimulated for 72 h with proinflammatory cytokines TNF-*α*, IL-6, IL-8, and IL-17F and the anti-inflammatory cytokine IL-4 (10 ng/mL). (a) IL-4 decreased RUNX2 gene expression at days 4 and 7, *n* = 7. (b) TNF-*α* and IL-6 decreased gene expression of COL1 at day 4 and 48 h, *n* = 7. (c) Only IL-4, but not the other cytokines tested, increased osteocalcin gene expression at days 4 and 7. Results are mean ± SD, *n* = 7. ^*∗*^Significant effect of cytokine treatment, *p* < 0.05.

The effect of treatment with the proinflammatory cytokines TNF-*α*, IL-6, IL-8, and IL-17F and the anti-inflammatory cytokine IL-4 (concentration of all cytokines tested 10 ng/mL) on osteogenic differentiation of hASCs was assessed. IL-4 significantly decreased RUNX2 gene expression by 4.7–5.0-fold at days 4 and 7 compared to untreated hASCs (Figure 2(a)).

COL1 gene expression was decreased by the proinflammatory cytokines TNF-*α* (4-fold decrease, day 4) and IL-6 (2.2-fold decrease, 48 h; Figure 2(b)). The other cytokines tested did not affect COL1 expression. IL-4 significantly increased the expression of the mature bone marker osteocalcin by 7.4-fold at day 4 and by 7.2-fold at day 7, compared to untreated controls (Figure 2(c)). The other cytokines tested did not significantly affect osteocalcin expression.

### 3.3. Pro- and Anti-Inflammatory Cytokines Enhanced ALP Activity and Mineralization of hASCs

**Figure 3:**
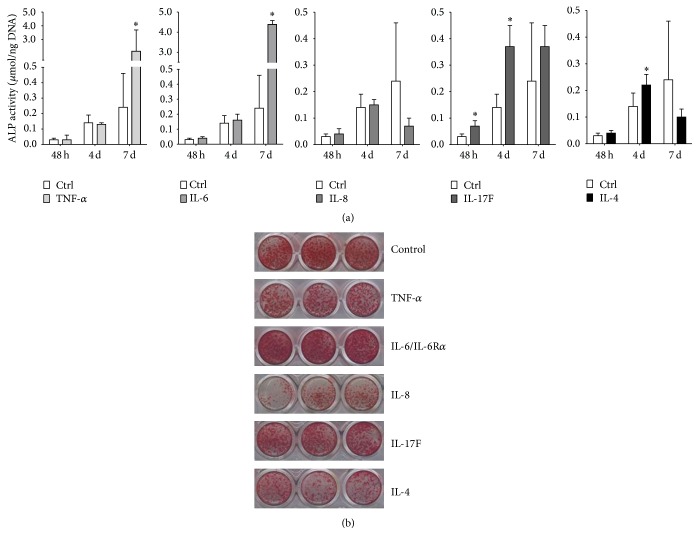
Comparative analysis of the effect of pro- and anti-inflammatory cytokines on osteogenic differentiation of hASCs. ASCs were stimulated for 72 h with proinflammatory cytokines TNF-*α*, IL-6, IL-8, and IL-17F and the anti-inflammatory cytokine IL-4 (10 ng/mL). (a) TNF-*α* and IL-6 increased ALP activity at 7 days and IL-17F at 48 h and day 7. IL-4 increased ALP activity at day 4. Results are mean ± SD, *n* = 3. ^*∗*^Significant effect of cytokine treatment, *p* < 0.05. (b) ASCs showed enhanced mineralization using alizarin red staining after IL-6 treatment at day 14 compared to untreated controls. TNF-*α*, IL-8, IL-17F, and IL-4 decreased mineralization of hASCs at day 14 compared to untreated controls.

The proinflammatory cytokines significantly increased ALP activity. TNF-*α* increased ALP activity by 9-fold and IL-6 by 18-fold at day 7. IL-17F increased ALP activity by 2.3–2.6-fold at 48 h and at day 4 (Figure 3(a)). IL-8 did not affect ALP activity at any of the time points measured. The proinflammatory cytokine IL-4 enhanced ALP activity by 1.5-fold at day 4 (Figure 3(a)).

IL-6 enhanced mineralization of hASCs at day 14 compared to the untreated controls. In contrast, TNF-*α* and IL-17F decreased mineralization of hASCs at day 14 compared to the untreated controls. Treatment with IL-8 and IL-4 resulted in low mineralization compared to untreated controls as well as compared to the other cytokines (Figure 3(b)).

## 4. Discussion

Understanding the mechanism of fracture repair, especially the inflammatory response, is relevant in the search for new strategies or treatments to optimize bone repair, which may have implications for the treatment of critical-size cranial defects. We added pro- and anti-inflammatory cytokines for 72 hours, which simulates the kinetics of their expression during early stages of fracture repair* in vivo*, and investigated their effects on the proliferation and osteogenic differentiation of hASCs.

The proliferative capacity of mesenchymal precursors is highly relevant for tissue repair [29]. Cytokines are known to affect proliferation of different cell types [22, 30]. Therefore, we first analyzed the effect of the different cytokines on the proliferation of hASCs. This study demonstrated that 10 ng/mL TNF-*α* increased DNA content of hASCs at 7 days. However, IL-4, IL-6, IL-8, and IL-17F (10 ng/mL) did not affect DNA content or expression of KI67. Thus, TNF-*α* may be more important than the other cytokines to induce MSCs proliferation during bone tissue repair. Interestingly, it has been reported that TNF-*α* at 50 ng/mL for 7 days does not affect MSC proliferation, while TNF-*α* at only 5 ng/mL significantly stimulates MSC proliferation by 2-fold [31]. We also showed that TNF-*α* at a relatively low concentration of 10 ng/mL increases hASC proliferation after 7 days of culture. It is thus possible that the observed effect of cytokines on indicators of stem cell proliferation, or lack thereof, is strongly dose-dependent.

Proinflammatory and anti-inflammatory cytokines differentially affected osteogenic differentiation of hASCs. TNF-*α* and IL-6 affected osteogenic differentiation of hASCs by decreasing COL1 gene expression, followed at a later stage by enhancing ALP activity. IL-6 also induced mineralization as shown by alizarin red staining of the cultures. Our findings confirm findings by others showing that IL-6 enhances osteogenic differentiation of MSCs [10, 32, 33]. Moreover, IL-6 at 100 ng/mL accelerates mineralization as well as RUNX2 gene expression in hASCs [28]. In our study, we used only 10 ng/mL IL-6 to treat hASCs, which might explain the lack of effect of IL-6 on RUNX2 expression. IL-6 has been shown to stimulate osteoblast differentiation [10]. A femoral fracture model in IL-6 knockout mice showed delayed callus remodeling and mineralization [32]. Therefore, IL-6 may play a crucial role in osteogenic differentiation of MSCs and might be used to enhance mineralization during fracture healing.

In the present study, 10 ng/mL IL-17F enhanced ALP activity by hASCs. In addition, the proinflammatory cytokine IL-17F stimulates osteoblast maturation and activation allowing bone synthesis [12, 14]. Four days of stimulation with IL-17F increases gene expression of COL1 and osteocalcin in MC3T3-E1 preosteoblasts and mouse primary mesenchymal stromal cells [12]. The difference between these data and our data might be related to differences in cell type and cytokine concentration, since we added IL-17F at 10 ng/mL to hASCs, while others added 20 ng/mL IL-17F to MC3T3-E1 preosteoblasts and mouse primary mesenchymal stromal cells [12].

Since our data showed that proinflammatory cytokines TNF-*α*, IL-6, and IL-17F affected the expression of proliferation and osteogenic differentiation markers by hASCs, we expected to also see an effect by IL-8 treatment. However, 10 ng/mL IL-8 did not affect proliferation or osteogenic differentiation of hASCs, suggesting that IL-8 does not likely play an important role in these processes during early stages of fracture repair. IL-8 is mostly known as an enhancer of cell migration, more than differentiation, and might thus still play a positive role in bone repair* in vivo*.* In vivo* studies are needed to unravel the role of IL-8 and the implications of its effects during the early stages of bone healing.

During fracture repair, the proinflammatory response switches to an anti-inflammatory response, where IL-4 may play an important role. Expression of the genes for T-cell effector cytokines such as IL-4 is significantly elevated in the fracture callus [34]. To our knowledge, this is the first study reporting on the effect of IL-4 on osteogenic differentiation of hASCs, although other cell types have been investigated [35–37]. A recent study has reported that bone marrow mesenchymal stem cells (BMMSCs) from FBN1-deficient (Fbn1^+/−^) mice exhibit decreased osteogenic differentiation and that this lineage alteration is regulated by IL4/IL4R*α*-mediated activation of mTOR signaling to downregulate RUNX2 [38]. So, this study provides relevant information that IL-4 is involved during osteogenic differentiation. In our study, we demonstrated that IL-4 at 10 ng/mL decreased gene expression of the early osteogenic marker RUNX2 but increased expression of the marker of later osteogenic differentiation osteocalcin in hASCs at days 4 and 7. IL-4 also increased ALP activity in hASCs. This is in agreement with findings by others showing that IL-4 stimulates ALP activity in a dose-dependent manner in cultured human osteoblasts and in the human osteosarcoma cell line MG63 [35–37]. On the other hand, we observed decreased mineralization in hASCs treated with 10 ng/mL IL-4, while others showed that M1 macrophages cocultured with preosteoblastic MC3T3 cells treated with IL-4 for 72 hours enhance osteogenic differentiation and mineralization [21]. MC3T3 monocultures treated with IL-4 for 72 hours did not reveal significant differences in mineralization compared with untreated MC3T3 cells [21]. This indicates that specific conditions within the inflammatory environment such as the presence of inflammatory cells, that is, macrophages, may influence the effects of IL-4 during fracture repair and then enhance the osteogenic differentiation of MSCs.

To obtain an optimal effect of cytokines in an* in vivo* critical-size cranial defect model is a significant challenge, since different concentrations of cytokines are produced, or different cytokine expression occurs by MSCs during their differentiation to osteoblasts [39]. The synergistic and antagonistic effects of different cytokines combined, as this occurs within the fracture site* in vivo*, are important, since the effect of combined cytokines might be different from the observed effect of individual cytokines on bone formation. We have focused on whether each cytokine will enhance or decrease the osteogenic potential of hASCs and on the time point that markers of bone formation are significantly expressed. A previous study from our group has also shown that the combination of cytokines present in the circulation of patients with active rheumatoid arthritis might contribute to generalized bone loss by directly inhibiting osteoblast proliferation and differentiation [40].

Exposure duration is also a critical element in determining cytokine effects on bone regeneration [41]. We showed that cytokines present during the inflammatory response may play an important role in the osteogenic differentiation of progenitor cells. Moreover, the coordinated interactions with cytokines, cells, and extracellular matrix have been documented to define a local biochemical and mechanical niche [42]. So, additional studies assessing the effect of proinflammatory and anti-inflammatory cytokines under conditions that better simulate the environment of the inflammatory response, such as hypoxia and the presence of inflammatory cells in a 3D environment, may provide additional information that might be useful when using MSCs and cytokines for bone tissue engineering purposes.

In summary, our data show that hASCs respond to the different cytokines by changes in osteogenic differentiation. Each cytokine analyzed had a specific effect in a specific time frame, which in combination with each other may enable successful bone repair. The stimulatory effects of IL-6 on ALP activity and mineralization in hASCs suggest that this cytokine may enhance osteogenic differentiation of MSCs and therefore could be used to optimize strategies focused on the treatment of critical-size cranial defects* in vivo*. In conclusion, all cytokines investigated seemed to exert both enhancing and reducing effects on osteogenic differentiation of hASCs. Specifically IL-6 may be suitable to induce osteogenic differentiation of MSCs as a strategy for enhancing bone repair.

## Figures and Tables

**Table 1:** Primer sequences for determination of proliferation and osteogenic differentiation of hASCs through PCR. *YWHAZ*: tyrosine 3-monooxygenase/tryptophan 5-monooxygenase activation protein, zeta; *UBC*: ubiquitin C; *KI67*: proliferation marker; *RUNX2*: runt-related transcription factor-2; *COL1*: collagen type 1; *Osteocalcin*.

Target gene (human)	Oligonucleotide sequences	
	Forward	Reverse
*YWHAZ*	5′ GATGAAGCCATTGCTGAACTTG 3′	5′ CTATTTGTGGGACAGCATGGA 3′
*UBC*	5′ GCGGTGAACGCCGATGATTAT 3′	5′ TTTGCCTTGACATTCTCGATGG 3′
*KI67*	5′ CCCTCAGCAAGCCTGAGAA 3′	5′ AGAGGCGTATTAGGAGGCAAG 3′
*RUNX2*	5′ ATGCTTCATTCGCCTCAC 3′	5′ ACTGCTTGCAGCCTTAAAT 3′
*COL1*	5′ TCCGGCTCCTGCTCCTCTTA 3′	5′ GGCCAGTGTCTCCCTTG 3′
*Osteocalcin*	5′AGCCACCGAGACACCATGAGA 3′	5′ CTCCTGAAAGCCGATGTGGTC 3′
